# *Eurosurveillance* reviewers in 2023

**DOI:** 10.2807/1560-7917.ES.2024.29.1.2401046

**Published:** 2024-01-04

**Authors:** Eurosurveillance editorial team

**Affiliations:** 1 European Centre for Disease Prevention and Control (ECDC), Stockholm, Sweden

**Keywords:** peer-review

The editorial team is grateful to all the experts who dedicated their time to peer-review our articles over the past 12 months. We wish to thank them and all those whose names are not listed below but gave helpful advice and support. We apologise to any reviewers whose names we may have missed.

Our reviewers in 2023 were:

Preben Aavitsland; Muna Abu Sin; Cornelia Adlhoch; Karoline Aebi-Popp; Seven Johannes Sam Aghdassi; Christina Ahlstrom; Ana Alastruey-Izquierdo; Bo Albinsson; Samuel Alizon; Vanessa Allen; Erik Alm; Brian Alper; Matthias an der Heiden; Maria an der Heiden; Fabrizio Anniballi; Richard Anthony; Alberto Antonelli; Patricia Antunes; Mardjan Arvand; Angel Asensio; Tatjana Avsic-Zupanc; Esteban Aznar; Anurima Baidya; Meghan Baker; Tamas Bakonyi; Sooria Balasegaram; Fausto Baldanti; Cyril Barbezange; Annalisa Bargellini; Ian Barr; Alexander Bartel; Jonathan Bastard; Ulrike Baum; Julien Beauté; Paul Beggs; Edward Belongia; Ria Benko; Beatrice Bercot; Nicole Berens-Riha; Jessica Beser; Carina Blackmore; Lauren Bloomfield; Pierre-Yves Boelle; Isaac Bogoch; Raquel Bonelli; Isabelle Bonmarin; Maria José Borrego; Jordi Borrell Pique; Thibault Bourdin; John M Boyce; Marieta Braks; Carina Brehony; Viviane Bremer; Nathan J Brendish; Eeva Broberg; Stefan Brockmann; Lynda Browning; Laura Bubba; Udo Buchholz; Christoph Buchta; Silke Buda; Nicholas Bundle; Luca Busani; Maria Bygdell; Merle Böhmer; Sindy Böttcher; Saverio Caini; Caterina Candela; Rafael Canton; Adam Capoferri; Heather Carleton; Yehuda Carmeli; David Carmena; AnnaSara Carnahan; Alessandro Cassini; Jesus Castilla; Vincent Cattoir; Simon Cauchemez; Orlando Cenciarelli; Meera Chand; Anuja Chatterjee; Saverio Checchi; Fanny Chereau; Gerardo Chowell; Paul Chua; Shuk Kwan Chuang; Jessie Chung; Hannah Chung; Nadia Ciampa; Cheryl Cohen; Michelle Cole; Brenda Coleman; John Conly; Marco Coppi; Marta Corbella; Victor Corman; Martin Cormican; Suzanne Cotter; Ana Paula Coutinho Rehse; Benjamin Cowling; Veronica Cristea; Angelo D'Ambrosio; Fortunato D'Ancona; Sanchita Das; Siddhartha Sankar Datta; Kerrie Davies; Irith De Baetselier; Irith De Baetselier; Brechje de Gier; Sigrid De Keersmaecker; Carmen de Mendoza; Gaston De Serres; Gerard de Vries; Tarik Derrough; Angel Desai; Aditi Dey; Vijaykrishna Dhanasekaran; Maureen Diaz; Liselotte Diaz Högberg; Sabine Diedrich; Frederika Dijkstra; Jo-Anne Dillon; Jac Dinnes; Gerhard Dobler; Katarzyna Domańska-Blicharz; Martin Dorner; Ann-Sofi Duberg; Jonathan Duffy; Jake Dunning; Peter Durr; Fernanda Dórea; Sarah Earnshaw Blomquist; Isabella Eckerle; Tim Eckmanns; Michael Edelstein; Roland Elling; Ehud Elnekave; Conrada Erkens; Mirko Faber; Massimo Fabiani; Daniel Faensen; Christopher Fairley; Gerhard Falkenhorst; Dennis Falzon; Paddy Farrington; Donna Ferguson; Helen Fifer; Julie Figoni; Antonietta Filia; Thea Fischer; David Fisman; Brendan Flannery; Ivo Foppa; Ron Fouchier; Leticia Franco; Christina Frank; Eelco Franz; Ingrid Friesema; Niels Frimodt-Møller; Ricardo Fuertes; Adam Gaelen; Amandine Gagneux-Brunon; Juan Carlos Galan; David García-García; Antonio Gasparrini; Tatjana Gazibara; Laura Giese; Marta Giovanetti; Jose Gonzales; Irene Gottgens; Mie Agermose Gram; Maria Grandahl; Donato Greco; Manfred Green; Laura Guerrero-Latorre; Vithiagaran Gunalan; Rebecca Guy; Stephan Göttig; Ágnes Hajdu; Ian Hall; John Halperin; Anette Hammerum; Lisa Hansen; Claudia Hanson; Heli Harvala; Rodrigo Hasbun; Henrik Hasman; Barbara Hauer; Anja Hauri; Joana Haussig; Sally Hayward; Louise Henaff; Antoni Hendrickx; Ana M Henriques; Edward Hill; Steen Hoffmann; Edward Holmes; Kathryn Holt; Charlotte J. Houldcroft; Torsten Houwaart; Torsten Houwaart; Jana Huisman; Yvan Hutin; Daniela Huzly; Ana Victoria Ibarra-Meneses; Thomas Inns; Giuseppe Ippolito; Kazuo Itabashi; Wiebke Jansen; Gerard Jansen; Elita Jauneikaite; Silvia Jimenez-Jorge; Kari Johansen; Pikka Jokelainen; Kai Jonas; Jefferson Jones; Maria João Cardoso; Annemarie Kaesbohrer; Suzanne Kalb; Tommi Karki; Yoshihiro Kawaoka; Roxanne Kerani; Gilbert Kersh; Robert Kirkcaldy; Freja Kirsebom; Esther Kissling; Wioleta Kitowska; Mirjam Knol; Maria Knoll; Philipp Kohler; Manuel Krone; Marcela Krutova; EJ Kuijper; Michael Kundi; Oliver Kurzai; Stefan Kuster; Jeff Kwong; Robin Köck; Massimo La Raja; Christoph Lange; Joanne Langley; Tinne Lernout; Vivian Leung; Kathy Leung; Bruno Lina; Adrian Lison; Pierluigi Lopalco; Claudia Lucarelli; Suzanne Lutgens; Frederik Lyngse; Theodore Lytras; Outi Lyytikäinen; Emma Löf; Christoph Lübbert; Kristine Macartney; Liane Macdonald; Gannon C.K. Mak; Georgia Mandilara; Anna Marchese; Isabella Marchesi; Ulrich Marcus; Inessa Markus; Gaetano Marrone; Melanie Marti; Esteban Martinez; Federico Martinón-Torres; Sébastien Matamoros; Wesley Mattheus; Leena Maunula; Jean Mbisa; Sarah McFarland; Matthew McGarrity; Angeliki Melidou; Pierrette Melin; Ella Mendelson; Gabriel Mendes; Stefano Merler; Kevin Messacar; Jesus Mingorance; Massimo Mirandola; Hana Mitchell; Neha Mittal; Reiko Miyahara; Amir M Mohareb; Hannah Moore; Orna Mor; Alain Moren; Joël Mossong; Laurent Moulin; Judith Mueller; Miguel Mulders; Zsófia Mészner; Priyanka Nannapaneni; Martha Nelson; Nathalie Nicolay; Vanja Nikolac Markic; Andreas Nitsche; Dorit Nitzan; Dorit Nitzan; Harold Noel; Isabel Noguer; Baltazar Nunes; Elisabet Nylander; Christiana Nöstlinger; Patrick O'Connor; Joan O'Donnell; Denise O'Sullivan; Ruth Offergeld; Akihiro Ohkado; Isabel Oliver; Ajibola Omokanye; Hana Orlíková; David Pace; W Paget; Romain Palich; Marcus Panning; Annalisa Pantosti; Anna Papa; Amy Parry; Lucia Pastore Celentano; Eleni Patsoula; Prabasaj Paul; Malik Peiris; Laura Pellegrinelli; Anne Perrocheau; Patrizio Pezzotti; Germán Peñalva; Nick Phin; Anne Piantadosi; Antonio Piralla; Adriana Pistol; Diamantis Plachouras; Christina Poethko-Müller; Anne Pohlmann; Laurent Poirel; Spyridon Pournaras; Joan Puig-Barbera; Joan Puig-Barbera; Natalia Rachwal; Benjamin Rader; Stéphane Ranque; Kaisu Rantakokko-Jalava; Agam K. Rao; Joren Raymenants; Carl Reddy; Giovanni Rezza; Hans Rieder; Nicole Ritz; Lucia Rivas; Annapaola Rizzoli; Lucy Robertson; Emmanuel Robesyn; Joacim Rocklöv; Maria Romay-Barja; Angela Rose; Gian Maria Rossolini; Pavitra Roychoudhury; Reiko Saito; Dan Salmon; Örjan Samuelsen; Frank Sandmann; Sabine Santibanez; Florian Saunier; Gianpaolo Scalia-Tomba; Henrieke Schimmel; Patricia Schlagenhauf; Daniel Schmidt; Axel Schmidt; Jonas Schmidt-Chanasit; Uffe Schneider; Oliver Schwartz; Eli Schwartz; Luigi Sedda; Alexandra Septfons; Luca Settanni; Ettore Severi; Giri Shankar; Abigail Shefer; Jenny Shen; Rosemary B. Sifford; Reina Sikkema; Lone Simonsen; Andreas Sing; Danuta Skowronski; Daniel Sobral; Ivan Solovic; Carla A. Sousa; Luigi Spagnolo; Gianfranco Spiteri; Ineke Spruijt; Shiranee Sriskandan; Molly K Steele; Paola Stefanelli; Gyde Steffen; Roger Stephan; Nicole Stoesser; Jennifer Stone; Sheena Sullivan; Vana Sypsa; Silvio Tafuri; Johanna Takkinen; Darrell Tan; Guyo Tao; Jacqueline Tate; Bernd-Alois Tenhagen; Daniel Thirion; Daniel Thomas; Nuria Torner; Mia Torpdahl; Alma Tostmann; Stephanie Totten; Florette Treurnicht; Karin Margareta Troell; Ruby S. M. Tsang; Maria Tseroni; Ákos Tóth; Soren Uldum; Taro Urase; Amra Uzicanin; Veronika Učakar; Janko van Beek; Wim Van Bortel; Marianne van der Sande; Marieke J. van der Werf; Catharina van Ewijk; Maaike van Mourik; Sonja van Roeden; Nina van Sorge; Thomas Vanwolleghem; Alkiviadis Vatopoulos; Fernando Vazquez; Tomas Vega-Alonso; Tomas Vega-Alonso; Pierre Verger; Ana Vieira; Leo Visser; Marie-Louise von Linstow; Chengming Wang; K Warriner; Atsuyuki Watanabe; Laurence Watier; Geoffrey Weinberg; Dirk Werber; Guido Werner; Heather Whitaker; Andreas Widmer; Kirsten Wiens; Lucas Wiessing; Mary Wikswo; Annelies Wilder-Smith; Hendrik Wilking; Christopher Williams; Maike Winters; Frank Wong; Elisa Wulkotte; Takuya Yamagishi; Askar Yedilbayev; Johanna Young; Hans L Zaaijer; Gernot Zarfel; Dominik Zenner; Michaela Špačková.

